# Physical Stress and Determinants of Shooting Performance Among Norwegian Special Forces Operators

**DOI:** 10.3389/fpsyg.2022.894169

**Published:** 2022-06-01

**Authors:** Jan Erik Buskerud, Frank Eirik Abrahamsen, Paul André Solberg

**Affiliations:** ^1^Department of Coaching and Psychology, Norwegian School of Sport Sciences, Oslo, Norway; ^2^Norwegian Olympic and Paralympic Committee and Confederation of Sports, Oslo, Norway

**Keywords:** shooting performance, marksmanship, SOF, military, combat, heart rate, physical stress

## Abstract

However, there is a lack of conceptual understanding of the factors influencing performance decrements in prone shooting. The present study examines how one can simulate a combat scenario by inducing acute physical stress, ultimately impacting one’s shooting performance (SP). The relationship between participants’ physical level and SP was measured in several ways. The SP of members of the Norwegian Navy Special Operations Forces (SOF) (*N* = 30) was measured before and directly after acute exercise-induced stress caused by a 200-m uphill run (90% HR_max_). Under acute physical stress, participants took less time to fire five rounds (total 15.5 ± 10.9 s faster), and the probability of hitting the target was unaffected (92%). In terms of more sensitive measures, score was significantly reduced and shot-group dispersion increased (64 ± 90 cm^2^, *p* < 0.01, *d* = 0.72), mainly due to increased vertical dispersion (2.5 ± 4.6 cm, *p* < 0.01, *d* = 0.53). Age, trait somatic anxiety and the Big Five Inventory item “openness” explained 45.2% of the variance in shooting score in the pre-physical stress condition. In the post-physical stress condition, pre-test shooting score, the number of months deployed, and shooting time predicted 32.9% of the variance in shooting score. The change in SP (pre—post) showed the concentration disruption scale was the best predictor of the reduction in shot score (20.1%). From a practical point of view, maintaining the probability of hitting the target with reduced shooting time post-physical stress could be viewed as superior performance for SOF.

## Introduction

Marksmanship is vital for soldiers’ survival, and therefore they need to control their behavior in challenging circumstances. Moving the muzzle 3 mm during the bullet strike leads to a deflection of as much as 25–30 cm over a 100-m distance (Chung et al., 2011). Shooting performance (SP) can be affected by many variables.

Chung et al. (2006) have conceptualized rifle marksmanship performance as a function of both skill and environment. While the environment is considered one-dimensional, the skill aspect consists of three *interrelated* components—cognitive, affective and perceptual–motor variables. Despite the importance of accurate shooting for different groups of people (e.g., sportsmen, police, biathletes, and military forces), there has been little effort to understand rifle shooting as a complex skill.

Special Operations Forces (SOF) have a physically and psychologically demanding occupation, and deployed military personnel have one of the most stressful professions in the world (Kensing, 2015). In military operations, soldiers are exposed to a multitude of stressors that potentially affect sensitive skills, such as shooting. One prominent stressor is physical stress. Soldiers often carry heavy equipment on missions (e.g., Knapik et al., 2004; Dean and DuPont, 2008). Loads of around 30 kg are common (e.g., Dean and DuPont, 2008), potentially increasing heart rate (HR) and ventilation (Jaworski et al., 2015), and resulting in muscle tremors (Lakie, 2010). All these things can affect the firing process, yet SP can quickly return to pre-exercise levels within a few minutes of physical stress (e.g., Evans et al., 2003; Frykman et al., 2012). However, taking a break is not always feasible, meaning that operators need to perform under suboptimal conditions. Consequently, the ability to quantify changes in performance under suboptimal conditions would be advantageous.

Stress and shooting from a standing position have been reasonably well researched (e.g., Nibbeling et al., 2014; Luchsinger et al., 2016; Tenan et al., 2017), but it is just as relevant to study shooting from the prone position. Prone shooting is often used when there is a need for precision, such as when shooting at a target from far away (sniping). Long-distance shooting is even more sensitive to deflections (Chung et al., 2011). Knowledge about stress and SP in the prone position will improve the understanding of operators working under various conditions.

To our knowledge, the study by Hoffman et al. (1992) is the only one that investigates SP before and directly after acute exercise-induced stress and that reports results for the prone position. Other researchers have studied combined performance measures for different shooting positions (e.g., Tharion et al., 1997; Tenan et al., 2017). These studies are important in terms of overall performance but lack the sensitivity to determine how stress affects individual shooting positions and when prone and standing shooting may be distinctively affected by physical stress. This distinguishing is important (Hoffman et al., 1992). Notably, the prone position is more stable compared to standing (Hoffman et al., 1992). Hoffman et al. (1992) examined SP of members of the US national biathlon team after exercise of different intensities. The probability of hitting a 4-cm target from a distance of 50 m did not change after exercise induced stress of increasing intensities, not even after peak maximal effort. Shooting scores and shot grouping only changed near the maximal effort.

A limitation of the study by Hoffman et al. (1992) was that shooting times were not recorded. Others have shown that taking more time could compensate for a decrease in SP for the standing position (Nibbeling et al., 2014). This might be relevant for prone shooting as well. Hoffman and Street (1992) found that the HR dropped as much as 50 beats/min during shooting for a duration of 50–60 s in simulated competitions (the starting HR was 87% HR_max_). A significant inverse correlation has been observed between HR and marksmanship in the standing position (Swain et al., 2011; Moore et al., 2014). Future research should therefore include temporal variables when investigating prone shooting, as time is critical in both combat and biathlon. Regarding SP in combination with physical stress, SP can either be affected directly by physiological variables (e.g., Duncan et al., 2013), or physical stress can function as a mediator of other important variables such as cognitive (e.g., McMorris et al., 2011) or psychomotor variables (e.g., Lyons et al., 2008). Several authors point to the effect of physical stress load with regard to shooting performance, but also that more training will increase the likelihood of performing well also during physical stress (e.g., Sánchez-Molina et al., 2018, 2019; Bustamante-Sánchez et al., 2020; Tornero-Aguilera et al., 2021).

Understanding the capacities of members of SOF has an important bearing on mission success, with psychological, ethical, economic, and practical implications. The aim of the present investigation was (a) to evaluate SP before and directly after physical stress and (b) to identify which variables best determine performance. It was hypothesized (a) that SOF members experience decreased SP on sensitive measures such as dispersion but maintain their shooting time because they are trained to respond quickly in combat and (b) that individual differences in HR will predict variations in SP.

## Materials and Methods

### Participants

Thirty male members of the Norwegian Navy Special Operations Command (NORNVASOC) participated in the experimental study (Table 1). All had completed a 2-year selection and training process that fewer than 10% of people pass. All were classified as expert shooters or sharpshooters.

**TABLE 1:** Characteristics of 30 SOF members participating in the study.

Variable	M ± *SD*
Age (years)	27 ± 4
Body height (cm)	184 ± 7
Body mass (kg)	87 ± 8
Experience (years)	7 ± 3
Shooting experience (years)	9 ± 6
Peak heart rate (beat ⋅ min^–1^)	196 ± 6
VO2 peak (mL ⋅ kg^–1^ ⋅ min^–1^)	61 ± 4

*VO2 peak was taken from internal tests in the NORNVASOC.*

Before providing written consent, participants received information about the potential risks of participating and were informed of their right to withdraw from the study. They were asked to limit their consumption of alcohol and caffeine and not to engage in intense physical activity 24 h before testing. The study was approved by the Norwegian Social Science Data Services and local military review boards.

### Study Design

This experimental study employed a within-subject design, an approach where the participants act as their own controls in order to see relative change. The participants were tested under two conditions, before and immediately after acute intense exercise. Testing consisted of firing three rounds of five shots to determine their pre-performance without any manipulated physical stress (pre-physical stress). They then undertook a 200-m uphill run and upon completion immediately picked up their rifles and fired another five shots (post-physical stress) in two separate series. Changes in SP, precision and shooting time were recorded. Sleep, activity, experience and psychological variables were measured using activity monitors and self-report questionnaires. Testing was conducted in three phases over 6 months to exclude potential seasonal variations and to ensure enough participants. All participants completed familiarization and testing procedures within 100 days before testing.

### Materials, Manipulation and Measures

#### Marksmanship Testing

Testing was conducted on a 100-m outdoor shooting range with individual lanes with participants unsupported in the prone position. The target was a circular black and white bullseye on a piece of paper. Pre-physical stress shooting trials consisted of firing three separate rounds of five shots. The use of paper targets prevented the participants from gaining visual feedback about the shots. After each trial, we photographed the targets for analysis before patching them.

Performance measures included the number of hits (%hit) and the shot score (points). The score ranged from 0 to 10 depending on the precision (60 cm diameter target with 3 cm between the scoring lines). % hit was defined as the percentage of successful hits inside the black circle (7 points or better, 24 cm diameter). The maximum possible shot score was therefore 50 points.

**FIGURE 1:**
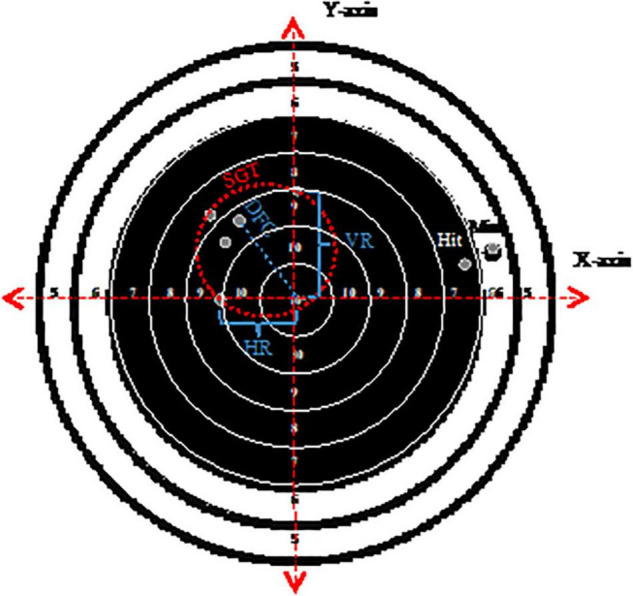
Illustrational overview over dispersion measures. Horizontal range (HR), vertical range (VR) and shot-group tightness (SGT) represents the correct values for the example shown. For distance from center (DFC) the example is only shown for one shot. The average for all five shots would be representative. Shots marked as Hit and Miss are added for extra illustrations. The circles from 4 to 0 points are not shown in the illustration.

When a bullet broke the line, the best score was given. For dispersion measures, the center of the bullet was used. The dispersion goals measured were horizontal range (HR), vertical range (VR), deviation from center (DFC), and shot-group tightness (SGT). Each shot was manually measured and given an x- and y-value with the target’s center points as the coordinate systems origo (Figure 1). The maximum range between the two most deviating shots on the x-axis represents the HR, and the same for the VR on the y-axis. DFC was calculated using the Pythagorean Theorem (a^2^ + b^2^ = c^2^), where the average for the five shots was reported in cm. SGT is the smallest circle fitting around all five shots (cm^2^), a dispersion measure used previously (Tharion et al., 1997). We designed a similar measurement program in Java with the help of an engineer.

#### Temporal Variables

Shooting time was recorded using the Pact Club Timer III (Pact Inc., Grand Prairie, TX, United States), where the shockwave from a shot is detected by the instrument. The timer was manually started when the shooter first touched their weapon. The time from start until the first shot (time to first shot) and the duration from the first shot to the last shot (time from first to last shot) was recorded.

#### Acute Exercise-Induced Stress

Exercise-induced stress (physical stress) was manipulated with a 200-m (about 1 min) steep up-hill run to the shooting range while wearing full gear (helmet, ear protection and bullet-proof vest, total 10–12 kg), except for the weapon for safety reasons. Based on studies by Hoffman and colleagues (Hoffman and Street, 1992; Hoffman et al., 1992) exercise intensity was set to 90% of the participants’ individual maximal HR. The percentage of HR maximum (HR_max_) excludes variations in HR_max_ and leads to an equal relative intensity.

#### Physiological Measures

HR was recorded continuously by a Polar H7 transmitter (Polar Electro Oy, Kempele, Finland) worn around the chest. Pre-HR was recorded when the participants took up their weapon and immediately after the last shot in each block (post-HR). The HR drop represents the pre-HR subtracted from the post-HR and was registered on an actigraph *via* Bluetooth with a 1-s registration rate. The actigraph was chosen because the participants should not be able to see their pulse in order to avoid behavioral changes (Hoffman and Street, 1992). With no information about their intensity, pre-testing familiarization was important to ensure correct intensity. Self-reported maximal HR was used as reference.

#### Rating of Perceived Exertion

We expected an increase in participants’ fatigue level to be accompanied by an increased rating of perceived exertion (RPE) and increased HR. Therefore, after each interval, participants rated their perceived exertion directly after shooting using a Borg scale ranging from 6 (no exertion) to 20 (maximal exertion) (Borg, 1982). The instrument has been reported to successfully measure RPE in previous studies (e.g., Pijpers et al., 2007; Blacker et al., 2013).

#### Self-Reported Questionnaires

##### Experience

Experience was reported using questionnaires asking about the number of years in the military, weapons, other weapon experience, number of deployments and other background information. Due to security reasons, some of this information is omitted.

##### Personality Trait

The Big Five Inventory (BFI) is one of the most accepted and commonly used questionnaires for testing personality traits (John et al., 1991), including reactivity to stress. BFI uses five broad dimensions to describe the human personality and psyche—extraversion, neuroticism, openness to experience, conscientiousness, and agreeableness. A Norwegian version of BFI-44 was used (Engvik and Føllesdal, 2005). The Cronbach’s alpha is shown to vary from 0.75 to 0.84, and in the present study it ranged from 0.60 to 0.85.

##### Multidimensional Trait Anxiety

Performance-related trait anxiety was reported using the Sport Anxiety Scale (SAS; Smith et al., 1990). A Norwegian version (SAS-N) was validated with a Norwegian sample (α = 0.75–0.88; Abrahamsen et al., 2006) and has been used among helicopter pilots in the military (Meland et al., 2015). SAS measures three sub-dimensions—somatic anxiety, worry and concentration disruption. In the present study, the alphas ranged from 0.60 to 0.86.

##### Multidimensional State Anxiety

Multidimensional state anxiety was measured immediately prior to the test protocol using the Mental Readiness Form-3 (MRF-3, Krane, 1994; Norwegian translation). The MRF is an abridged alternative to the popular CSAI-2 (Martens et al., 1990) with reports of moderate to excellent correlations between CSAI-2 and MRF-3 (0.68–0.76; Krane, 1994). MRF consists of one statement for three subscales—cognitive anxiety, somatic anxiety, and self-confidence. The Cronbach’s alpha revealed good reliability for the three subscales together (0.90).

##### Perceived Competence

Perceived competence was measured with a translated and shooting-adapted version of the four-item Perceived Competence Scale (PCS; Williams and Deci, 1996). The original scale has been tested in several studies (Williams et al., 2004, 2006), and a Norwegian translation has shown acceptable reliability (α > 0.90; Solberg et al., 2013). The Cronbach’s alpha was adequate (0.87).

#### Sleep and Activity

Sleep and activity were recorded 24 h before the test protocol using ActiGraph GT3X-Bluetooth (Pensacola, FL, United States). The device is used in a number of epidemiological studies, reporting reliable measures of sleep and activity (e.g., Santos-Lozano et al., 2013). GT3X is a small device (4.6 cm × 3.3 cm × 1.5 cm and 19 g) worn like a watch on the non-dominant hand and does not affect daily activity. Data were logged for analysis with ActiLife software 6.13.3 (ActiGraph). The validated Cole–Kripke algorithm was used to estimate sleep Cole et al. (1992), and the Fredson combination algorithm was used to estimate activity (Fredson et al., 1998). Energy consumption was only recorded during activity; the basal metabolic rate was not included.

### Pilot and Pre-testing Familiarization

Pilot testing was performed 2 months before the first test round. It was imperative to have a simple and sensitive test to be able to investigate the separate impact of physical stress. Various shooting approaches were tested (distances, weapon, targets, shooting range) in the pilot. Three stress trials were completed during the pilot. Some participants experienced difficulty reaching 90% HR_max_ in all three trials. To ensure correct intensities, one trial was removed from the test protocol. Three pre-physical stress trials were still considered acceptable.

### Procedures

Participants started wearing the sleep and activity monitor a minimum of 24 h before the start of the test protocol. The actigraph device was initialized with 1-s epochs and registration at 100 Hz to improve the sensitivity. When the participants arrived on the test day, all actigraph monitors were collected and the data downloaded. The device was again initialized with the same registration rate, but Bluetooth was now activated to log HR data for offline analyses. All participants gave written informed consent and answered all questionnaires. Magazines were then loaded, and zeroing began after going through the test procedure and safety instructions. Twenty min were allowed for the zeroing due to the individual needs identified in the pilot. It was essential to have enough time and avoid stressful conditions for the zeroing to ensure correct sight adjustments and valid shooting results.

After zeroing, no further adjustments were allowed. Before pre-physical stress registration,[Frame1] the participants were instructed to shoot the three trials (five shots) as fast as possible without sacrificing precision. All trials were performed in plenum on command (ready, fire). Next, a physical warm-up was conducted that consisted of 15 min of jogging with increasing intensity (60–75% HR_max_). Pre-testing familiarization ensured that all participants maintained correct intensity (90% HR_max_). Participants started individually with 1 min between each participant. The uphill run proceeded and finished on the shooting range; the weapons were picked up on arrival. Perceived exertion was reported after five shots. A 10-min break followed, with a low-intensity walk back to the start. The whole procedure was repeated once. All testing was performed in temperatures between 5^°^ and 8^°^ with 70–85% humidity between 12:00 and 3:00 p.m.

### Statistics

All statistical analyses were performed in SPSS, version 24 (IBM Corporation, Armonk, NY, United States). Normality assumptions were checked using Shapiro–Wilk tests, and all reported data were found to be normally distributed. Means ± standard deviations were calculated for all psychological variables (Table 2). Repeated *t*-tests for independent samples were employed to analyze differences between shooting scores in pre- and post-stress conditions. No differences were detected in shooting variables between the three pre- and the two post-stress tests. Only the mean values for the pre- and post-stress variables are presented and used for further analysis (Table 3). Mean values for pre- and post-stress variables were checked for differences using independent *t*-tests. Effect sizes were calculated using Cohen’s ***d*** (1988) with values 0.2, 0.5, and 0.8 representing small, moderate and large, respectively. Two-tailed Pearson’s correlations were used to assess the relationship between mean values for pre-stress, post-stress and change in shot score for all related variables. Finally, to explain the variance in shooting scores for the three conditions, we used multiple regression analysis based on significant correlations from the Pearson’s tests (enter). Statistical significance was set at *p* < 0.05.

**TABLE 2:** Mean ± standard deviation for Big Five Inventory personality items, perceived competence, trait- and state anxiety among the participants.

Variable	*M* ± *SD*
**Big five inventory items**	
Extraversion (8–56)	38.8 ± 7.2
Agreeableness (9–63)	47.7 ± 4.1
Conscientiousness (9–63)	48.3 ± 4.7
Neuroticism (8–56)	20.0 ± 5.8
Openness to experience (10–70)	43.9 ± 7.1
**Sport anxiety scale-n (SAS)**	
Somatic anxiety (1–4)	0.8 ± 0.3
Worry scale (1–4)	0.8 ± 0.3
Concentration disruption scale (1–4)	1.3 ± 0.3
**Mental readiness form (MRF)**	
Cognitive anxiety (1–11)	2.7 ± 1.5
Somatic anxiety (1–11)	2.9 ± 1.5
Self-confidence (1–11)	8.0 ± 1.4
Perceived competence scale (PCS) (1–7)	5.0 ± 1.6

**TABLE 3:** Mean ± standard deviation values for shooting performance, dispersion, temporal variables, rating of perceived exertion and heart rates for the three pre-physical stress tests and two post-physical stress tests.

Variable	Pre-stress (M ± *SD*)	Post-stress (M ± *SD*)	Effect size (Cohen’s *d*)
**Performance variables**			
%Hit	92 ± 14	92 ± 11	0.08
Points (0–50)	45 ± 3	41 ± 4*	0.88*
**Temporal variables**			
Time to first shot (s)	22.5 ± 8.5	13.6 ± 7.4**	0.74**
Time from first to last shot (s)	21.6 ± 11.7	15.0 ± 6.7*	0.40*
**Dispersion variables**			
Shot group tightness (cm^2^)	68.8 ± 40.6	133.2 ± 97.0**	0.72**
Distance from center (cm)	5.5 ± 2.0	6.9 ± 2.1	0.54
Horizontal range (cm)	11.1 ± 18.8	9.4 ± 4.2	0.08
Vertical range (cm)	7.5 ± 2.5	10.0 ± 4.7**	0.53**
**Heart rate**			
Pre-shooting heart rate (%)	46 ± 10	90 ± 4**	0.66**
Heart rate drop (beats ⋅ min^–1^)	5 ± 15	19 ± 9	0.19
**Rating of perceived exertion**			
Borg scale (6–20)		17 ± 1	

*Indicates significant difference between pre- and post-physical stress at P < 0.05 and **P < 0.01. Rating of perceived exertion (Borg scale) was only registered post-stress. Pre-shooting heart rate is measured. When grabbing the weapon and moving into the prone shooting position.

## Results

### Sleep and Activity

Participants walked 16,294 ± 3,669 steps, with average energy expenditure of 2,331 ± 610 kcal in the last 24 h before testing. Sleep data revealed the participants spent 6 ± 2 h and 25 min in bed with sleep efficiency at 89 ± 6%. During the sleep period, the participants experienced an average of 16 ± 6 awakenings.

### Psychological Measurements

All psychological measurements are presented in Table 2.

### Effects of Physical Stress

The manipulation led to a PRE of 17 (very hard) and HR at 90% of max, which was significantly higher than pre-testing but did not change significantly during shooting (Table 3). More physical stress produced no change in the number of hits (%Hit), but the shot score (points) decreased compared to the pre-stress condition. Dispersion measures indicated a significantly larger shot-group dispersion with an increase of as much as 194% in areal size. Vertical range was mostly affected by inducing stress, while horizontal range surprisingly showed a tendency toward a lower amount of sideways dispersion. Temporal characteristics changed significantly under physical stress compared to baseline. Participants took significantly less time to fire the first shots and to complete the five shots.

### Multiple Regression Analysis

Multiple regression analysis (MRA) was used to determine the variance in SP, examining what best predicted the shot score for the pre- and post-physical stress conditions and the changes in shot score. Regression analyses indicated the BFI item “openness to experience,” age and SAS somatic anxiety explained 45.2% of the variance in shot score for the pre-physical stress condition (Table 4). Collinearity statistics did not indicate significant multicollinearity among the predictive variables [tolerance 0.968–0.995, variance inflation factor (VIF) 1.005–1.033]. For the post-physical stress condition, shot score pre-physical stress, the shooting time from the first to the last shot and the number of deployments (months) explained 32.9% of the variance in shooting score after acute physical stress (Table 5; tolerance 0.852–0.943, VIF 1.061–1.173). Regarding changes in shot score from the pre-physical stress condition to the post-physical stress condition (Δ shot score), SAS “concentration disruption” was the only significant predictor, accounting for 20.1% of the decrease in shooting score (Table 6).

**TABLE 4:** Summary of analysis of multiple linear regression for the pre-physical stress condition.

Independent variable	*b*	*t*	*p*
Shot-core pre-stress (constant)	34.287	7.048	0.00
Big Five openness to experience	–0.144	–0.345	0.020
Age	0.390	3.495	0.002
SAS somatic anxiety	3.433	2.711	0.012

*b, unstandardized beta coefficients.*

**TABLE 5:** Summary of analysis of multiple linear regression for the post-physical stress condition.

Independent variable	*b*	*t*	*p*
Shot-score post-stress (constant)	24.208	2.714	0.012
Time from first to last shot	–0.205	–2.396	0.024
Pre-stress points	0.436	2.182	0.038
Deployments	0.208	1.832	0.078

*b, unstandardized beta coefficients.*

**TABLE 6:** Summary of analysis of multiple linear regression for change in shooting score.

Independent variable	*b*	*t*	*p*
Δ Shot-score (constant)	–5.972	–1.849	0.075
SAS concentration disruption scale	6.614	2.882	0.008

*b, unstandardized beta coefficients.*

## Discussion

The present study examined whether the SP of experienced SOF members would be affected by acute physical stress. The results revealed that although HR increased to 90% of HR_max_, the probability of hitting the target was unchanged (%Hit), and the participants used significantly less time. More sensitive measures, such as points, SGT and VR, revealed significant changes. The results confirmed our hypothesis that SOF members would experience a drop in SP on sensitive measures when not compensating by extending shooting time.

The present study shows that experienced members can maintain their probability of hitting the target in physically stressful conditions; these findings are similar to those of previous research (e.g., Hoffman et al., 1992; Bustamante-Sánchez et al., 2020). Sensitive information about shooting dispersion is vital because it provides insight into both the degree and direction of any changes. In the study by Hoffman et al. (1992), shot score and group diameter only changed significantly for the peak condition, but a linear tendency was observed with increasing intensities. Hoffman et al. (1992) did not report any direction for their dispersion (horizontal and vertical). In our study, a larger vertical range explained the increased group dispersion. In the prone position, filling the lungs with air mainly leads to vertical movement of the weapon. The link between SP and ventilation rate is significant after acute exercise (Moore et al., 2014). Increased intensities may result in more frequent and greater ventilation, likely causing increased vertical dispersion.

Additionally, ventilation might affect temporal variables. For SOF members, response time may be as important as accuracy. Our results suggest that participants decreased both the time to take the first shot and the time to complete the five shots in the post-physical stress condition compared to the pre-physical stress condition. A limitation of the study by Hoffman et al. (1992) is that they did not report shooting times, thus decreasing the possibility for appropriate comparison. Biathletes from the same group (the US national team) were tested the same year during simulated competitions. Their results revealed that the biathletes took 50–60 s on the prone shooting (five shots) with HR at 87% (166 beats per min) at entrance (Hoffman and Street, 1992). During the 50–60 s shooting period, HR dropped approximately 50 beats per min. In comparison, the HR of the participants in the present study dropped approximately 20 beats per min during the 28.6 s period. If similar shooting times and the HR drop is relevant for Hoffman et al. (1992) it might explain why their athletes only experienced a significant reduction at peak performance. Taking a longer time at the shooting range may reduce shooters’ HR. HR has shown a significant inverse correlation with marksmanship (Swain et al., 2011), and additional aiming time might compensate for a drop in SP (e.g., Nibbeling et al., 2013).

We hypothesized that HR would predict SP, but this was not the case for either condition. In contrast, the post-physical stress shooting score and shooting time from the first to the last shot had a significant inverse relationship. This could mean that taking less time was related to a better shooting score post-physical stress because increased ventilation makes correct and fast timing even more important. It could also be that because our participants are highly trained to respond quickly in stressful situations the experiment was not sufficient to alter their performance. Reaction time is often even more important than precision, especially for close-range shooting. The regression analyses indicated that deployments and shooting score pre-physical stress significantly explained the variance in SP, highlighting the relevance of rigorous training.

First, our results indicate that it is advantageous to have a high baseline shooting level. If special forces operators can constantly hit the target without any stress, they are probably more robust under physically stressful conditions. Second, SOF members with more real-life experience (deployments) perform better while physically stressed compared with their less experienced peers. The results might indicate that our testing is practical and relevant because the most experienced members performed better, somewhat in line with the findings of for instance Sánchez-Molina et al. (2019).

SOF are highly trained and members are selected based on their psychological characteristics. In general, the SOF members had very low BFI scores for “neuroticism” and both anxiety measures (state and trait). Based on their psychological characteristics (indicating a low probability of being psychologically stressed), it might be that physical stress has more impact on SP in combat scenarios because the more experienced members performed better in the post-physical stress condition. Physiological variables did not indicate any relationship with performance in the present study.

For the pre-physical stress condition, however, other variables explained the variance in shooting score. The BFI item “openness to experience,” age and SAS somatic anxiety were all significant predictors. Somatic anxiety and age revealed a positive relationship whereas openness to experience was negatively correlated. While a positive correlation with age is more self-explanatory, the two other variables are not that obvious.

State anxiety is traditionally associated with SP (e.g., Sade et al., 1990). In our results, somatic trait anxiety had an impact on pre-physical stress SP, but no dimensions of state anxiety reached significance. It may be that our instrument for measuring state anxiety, MRF, was not sensitive enough to detect individual variations (floor effect).

One question that requires further examination is whether higher trait anxiety spurs skilled SOF members to focus on development daily, whereas in real settings they exhibit relatively low state anxiety. In other words, they might worry for the future but stay in the moment during stress. A limitation in our study is that we did not document coping strategies, which may function as mediators. In addition, some of the constructs in SAS have been criticized (e.g., Abrahamsen et al., 2006).

Openness was the final explanatory variable in the pre-physical stress analysis. It is used to describe a person’s curiosity, intellect, creativity, and divergent thinking, that is, whether a person is open to new feelings and ideas and is willing to use their imagination. While there might not be any apparent link between openness and sport performance (e.g., Teshome et al., 2015), people scoring high on openness tend to prefer jobs that involve a high degree of creative thinking (e.g., Ozer and Benet-Martínez, 2006) and situations where one must continuously adapt to changes (e.g., Raudsepp, 1990). Flexibility is essential for SOF members during changing mission circumstances.

By examining the openness subscale more closely, we found discrepancies between low scores on fantasy, aesthetics, and feelings and higher scores on action, curiousness and ideas. Being creative and solution-oriented could be essential to improve learning and development (testing new ideas), eventually helping SP in the long run.

The last regression analysis examined the relationship between the reduction in shot score from pre- to post-physical stress and the SAS concentration disruption scale. The analysis was significant and is logical at the outset. Those who are exhausted after running might have lower concentration and experience a performance drop; However, this result must be reviewed carefully, as the concentration disruption scale had relatively low internal consistency.

Unfortunately, studies examining concentration, SP and physical stress are almost non-existent. Luchsinger et al. (2016) investigated brain activity measured with EEG before and after vigorous exercise in a laboratory setting. The results indicated that frontal theta activity (4–7 Hz) associated with focused attention was different among experienced and inexperienced biathletes. Athletes in that study did not experience a drop in performance pre- vs. post-stress, so to what degree focused attention would predict a decrease in performance after physical stress remains unknown and requires further investigation.

In future research, it would be of interest to explore to a deeper extent what physical stress affects SP at different skill levels and to understand which variables explain level variations. Mechanical, physiological, and psychological effects should all be with greater nuances to understand the dynamics between these variables in relation to marksmanship performance. It is highlighted that future research should include temporal variables and sensitive shooting measurements to detect potential performance changes.

This study have some limitations that. It is a strength that groups of high-performance practitioners often replicate steady performances, but a disadvantage is the sheer number of possible participants. Statistically significant results are harder to achieve with homogeneous participants, and the results might not be generalizable. This constitutes the first limitation.

The second limitation is that the results from a simulated physical stress setting with a non-moving target might not be representative of real-life situations. It is more important that the actual performance in the line of duty is superior.

The third limitation is that while our intentions were to control and adjust for several possible confounding factors, the broad approach might negate the sensitivity of the study. We prioritized short questionnaires with high reliability over other valid instruments, but there are several limitations with this. For example, self-report scales are not as sensitive as physiological measures. To what extent self-report questionnaires measure somatic anxiety is questionable. In retrospect, we should probably have included an instrument for concentration in the performance setting and for coping strategies.

The present study has implications for the military, the police and the Olympic sport of biathlon. Our data suggest that special forces operators SP can tolerate acute physical stress. From a practical point of view, hitting the target with little change in accuracy while taking less time represents superior performance and might be life-saving. In essence, it seems that the dispersion in the vertical range was most sensitive for increased physical stress. For police and military personnel, vertical dispersion is usually not as critical as it is for biathletes who use a circular target. This study shows that shooters with a good non-stress level are more robust in stressful situations.

## Conclusion

In conclusion, our study shows that NORNAVSOF members were able to maintain their probability of hitting a target in the prone shooting position while being physically stressed (90% HR_max_). Regarding the more sensitive measures, shot score, shot-group tightness and vertical dispersion changed with faster shooting times. Shooting time explained individual variations in shooting score in the post-physical stress condition. In contrast to the existing literature, superior performance was associated with taking less time.

## Data Availability Statement

The raw data supporting the conclusions of this article will be made available by the authors, without undue reservation.

## Ethics Statement

The studies involving human participants were reviewed and approved by the Norwegian Social Science Data Services and Local Military Review Boards. The participants provided written informed consent to participate in the study.

## Author Contributions

JB, PS, and FA were involved in the conceptualization and design of this study. JB and PS collected and analyzed the data and interpreted the results. JB drafted the initial manuscript with constructive feedback in the process. All authors read and approved the final manuscript.

## Conflict of Interest

The authors declare that the research was conducted in the absence of any commercial or financial relationships that could be construed as a potential conflict of interest.

## Publisher’s Note

All claims expressed in this article are solely those of the authors and do not necessarily represent those of their affiliated organizations, or those of the publisher, the editors and the reviewers. Any product that may be evaluated in this article, or claim that may be made by its manufacturer, is not guaranteed or endorsed by the publisher.
